# Intelligent localization and quantitative evaluation of anterior talofibular ligament injury using magnetic resonance imaging of ankle

**DOI:** 10.1186/s12880-021-00660-x

**Published:** 2021-08-28

**Authors:** Wen Yan, Xianghong Meng, Jinglai Sun, Hui Yu, Zhi Wang

**Affiliations:** 1 School of Precision Instrument and Opto-Electronics Engineering, Tianjin University, Nankai District, 92 Weijin Road, Tianjin, 300072 China; 2 Radiology Department, Tianjin Hospital, 406 Jiefangnan Road, Hexi District, Tianjin, 300210 China

**Keywords:** Anterior talofibular ligament, Intelligent localization of ATFL, Quantitative evaluation of ATFL injury, DRLSE, Magnetic resonance imaging

## Abstract

**Background:**

There is a high incidence of injury to the lateral ligament of the ankle in daily living and sports activities. The anterior talofibular ligament (ATFL) is the most frequent types of ankle injuries. It is of great clinical significance to achieve intelligent localization and injury evaluation of ATFL due to its vulnerability.

**Methods:**

According to the specific characteristics of bones in different slices, the key slice was extracted by image segmentation and characteristic analysis. Then, the talus and fibula in the key slice were segmented by distance regularized level set evolution (DRLSE), and the curvature of their contour pixels was calculated to find useful feature points including the neck of talus, the inner edge of fibula, and the outer edge of fibula. ATFL area can be located using these feature points so as to quantify its first-order gray features and second-order texture features. Support vector machine (SVM) was performed for evaluation of ATFL injury.

**Results:**

Data were collected retrospectively from 158 patients who underwent MRI, and were divided into normal (68) and tear (90) group. The positioning accuracy and Dice coefficient were used to measure the performance of ATFL localization, and the mean values are 87.7% and 77.1%, respectively, which is helpful for the following feature extraction. SVM gave a good prediction ability with accuracy of 93.8%, sensitivity of 88.9%, specificity of 100%, precision of 100%, and F1 score of 94.2% in the test set.

**Conclusion:**

Experimental results indicate that the proposed method is reliable in diagnosing ATFL injury. This study may provide a potentially viable method for aided clinical diagnoses of some ligament injury.

## Background

There is a high incidence of injury to the lateral ligament of the ankle in daily living and sports activities [1, 2]. The incidence was one case per 10,000 person-day in the world, ranking first in trauma emergency cases [3]. The anterior talofibular ligament (ATFL) is the most frequent types of ankle injuries [4]. It is the connective tissue connecting the fibula to the talus, which is vital for maintaining the stability of the ankle [5]. Different severity of injury corresponds to different treatment and prognosis. Therefore, it is of great significance to accurately judge the severity and choose appropriate treatment methods for reconstructing the stability of ankle and preventing repeated sprain. At present, the diagnosis of ATFL injury is mainly based on magnetic resonance imaging (MRI) [6], and relies on doctors' extensive reading of MR images. Moreover, it requires high clinical experience for doctors, and the diagnosis from different doctors can also be controversial. There is an urgent need to develop an intelligent diagnosis method for the patients with ATFL injuries, detecting the injuries automatically, accurately, and immediately either in the acute phase or during rehabilitation [7].

MRI has the advantage of high soft tissue resolution, which can clearly display anatomical details such as ligaments and tendons [8]. It has been routinely applied in the examination of ankle injury [9]. However, qualitative evaluation using MRI cannot dynamically evaluate ligaments and provide quantitative morphological features to guide diagnosis, treatment and prognosis.

The ATFL tear shows some qualitative changes on MRI. The ATFL loses normal tension and appears as a wavy or curvilinear abnormality on the images [10]. The contour is irregular and blurry. Compared with the low signal of normal tendons in the same slice, there are obvious high signal shadows like cords and patches in the ATFL [11]. Nevertheless, there are still few studies on quantitative analysis of ATFL tear, and no definite quantitative criteria can be used for clinical diagnosis. Liu et al. [12] found that the ligament thickness and signal to noise ratio of ATFL patients were significantly higher than those of the control group. Basha et al. [13] proposed that the bright rim sign was a very helpful diagnostic sign in assessment of ATFL disruption. Mun et al. [14] concluded that the sensitivity, specificity and accuracy of diagnosis using the cross-sectional area of the ligament were higher than the ligament thickness. These findings suggested that morphological measurement based on MR images is of predictive value in the diagnosis of ATFL tear. However, these features were all manually marked and measured by doctors, which is laborious and subjective. There is no automatic localization and segmentation algorithm of ATFL, and the related research is limited. A semiautomatic anterior cruciate ligament segmentation program that utilized morphological operations and active contour was proposed [15], which shows a low performance with Dice coefficient of 38.1%. Vinay et al. [16] used hybrid level set active contour to segment anterior cruciate ligament, but the poor results indicated that much improvement is still necessary before the application of clinical image diagnosis. Flannery et al. [17] developed an automated segmentation method for the anterior cruciate ligament, and the final model scored well on anatomical performance metrics (Dice coefficient = 0.84, precision = 0.82, and sensitivity = 0.85). However, there is still no relevant lesion localization and segmentation model for ATFL.

To improve the above defects, an intelligent localization method based on the priori knowledge of ATFL was proposed in this study. The key slice can be obtained by extracting the foreground contour and the central bone contour from MRI cross-sectional images. According to the anatomical characteristics of talus and fibula, feature points can be found to locate ATFL area. Then, the first-order gray features and second-order texture features of ATFL area were quantified for evaluation of ligament injury. The presented method does not require too much data, and only requires the combination of doctors' prior knowledge and image processing algorithm. Furthermore, these topics including the key slice extraction, lesion automatic localization and intelligent evaluation in this paper are a new attempt for the evaluation of the ATFL injury, which is of great significance for clinical diagnosis.

## Methods

### Datasets

In this study, data were collected retrospectively for patients with acute ankle sprain admitted to Tianjin Hospital, Tianjin, China from January 2019 to January 2020. The inclusion criteria were patients who (1) had a history of acute ankle sprain injury; (2) had positive clinical findings suggestive of ligamentous injury; and (3) had complete imaging data. The exclusion criteria were patients who (1) had a history of recurrent ankle sprain injury; (2) had a history of ankle joint infection, deformity, fracture, tumor and surgery; and (3) had incomplete imaging data or blurred images. According to the above inclusion and exclusion criteria, a total of 158 ankle sprain patients were studied, aged from 13 to 76 years old. There were 68 (43%) patients in the normal group and 90 (57%) in the tear group for ATFL, of which 64 patients were partial tear and 26 were complete tear. The basic characteristics of patients are presented in Table 1. The t-test results show that no significant statistical characteristics are detected between the normal and tear groups (*P* > 0.05).

**Table 1:** The basic characteristics of patients

Characteristics	Total (n = 158)	Normal (n = 68)	Tear (n = 90)	*P*
Age (y), mean (range)	33.0 (13–76)	35.7 (13–68)	31.0 (14–76)	0.338
Sex, Male, n (%)	94 (59)	40 (59)	54 (60)	0.882
Sex, Female, n (%)	64 (41)	28 (41)	36 (40)	
Side, Left, n (%)	77 (49)	30 (44)	47 (52)	0.316
Side, Right, n (%)	81 (51)	38 (56)	43 (48)	

MRI was obtained from the radiology department. All patients had an MRI scan of the ankle with a 3.0-T magnet (Ingenia CX, Philips Healthcare, Best, the Netherlands). The scan ranges from the distal tibia to upper calcaneus. The sequences include cross-sectional T2 weighted images with fat suppression, sagittal T1 weighted images, sagittal proton density weighted images with fat suppression and coronal proton density weighted images with fat suppression. Since the diagnosis of ATFL injury is mainly based on cross-sectional images, this study focused on the cross-sectional T2 weighted images with fat suppression (TR 4,000 ms, TE 71 ms, FOV 180 × 180 mm, slice thickness 4 mm, and spacing between slices 0.4 mm).

Results were classified as normal, partial tear, or complete tear as described by Joshy et al. [18]. Partial tear was defined as partial adhesion of the ligament fibers and a coarse cut fibre surface with intact continuity. It was characterized by the thickening and tortuosity of the ligament, and the discontinuity of some fibers. Complete tear was defined as entire discontinuity of the ligament and adhesion of adjacent tissue [19, 20]. The focal localization and diagnostic results of the ATFL in all patients were labeled independently by two radiologists. For cases with inconsistent or controversial labeling results, an experienced associate chief physician will review and discuss the consultation to reach an agreement. Also, the radiologists have completed training for musculoskeletal radiology at Tianjin Hospital, China, which is a comprehensive hospital specializing in orthopedics.

### Structure of this study

**Fig. 1:**
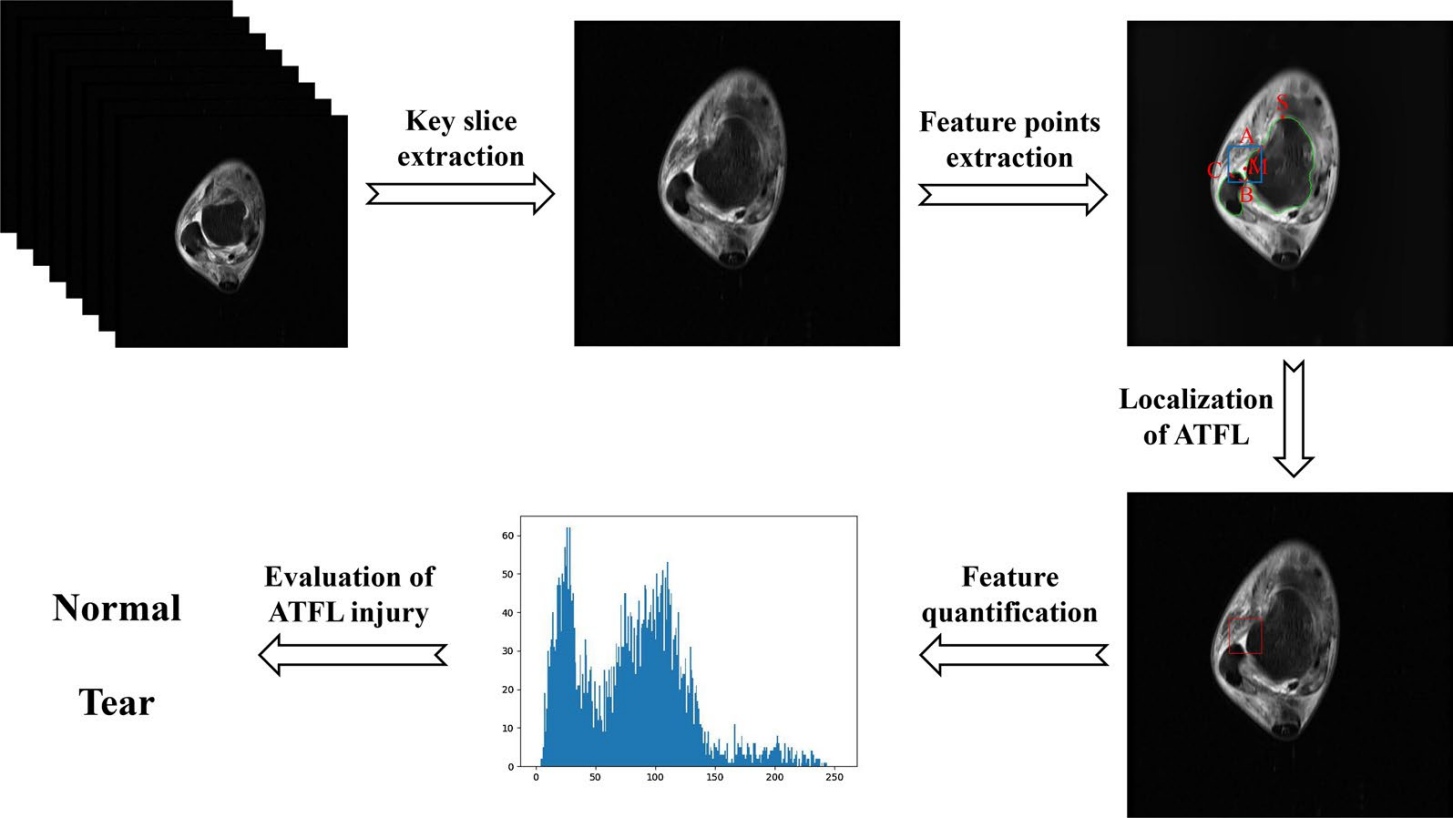
The structure diagram of this study

As shown in Fig. 1, the aim of this work is to achieve intelligent localization and quantitative evaluation of the ATFL. First, the slice containing the ATFL was extracted from cross-sectional T2 weighted images with fat suppression of ankle joint. Then, according to anatomical structure characteristics of the talus and fibula in the key slice, the contours and the feature points were extracted to locate the ATFL area. Subsequently, the first-order gray features and second-order texture features were calculated for evaluation of ATFL injury.

### Intelligent localization of ATFL

#### Key slice extraction

**Fig. 2:**
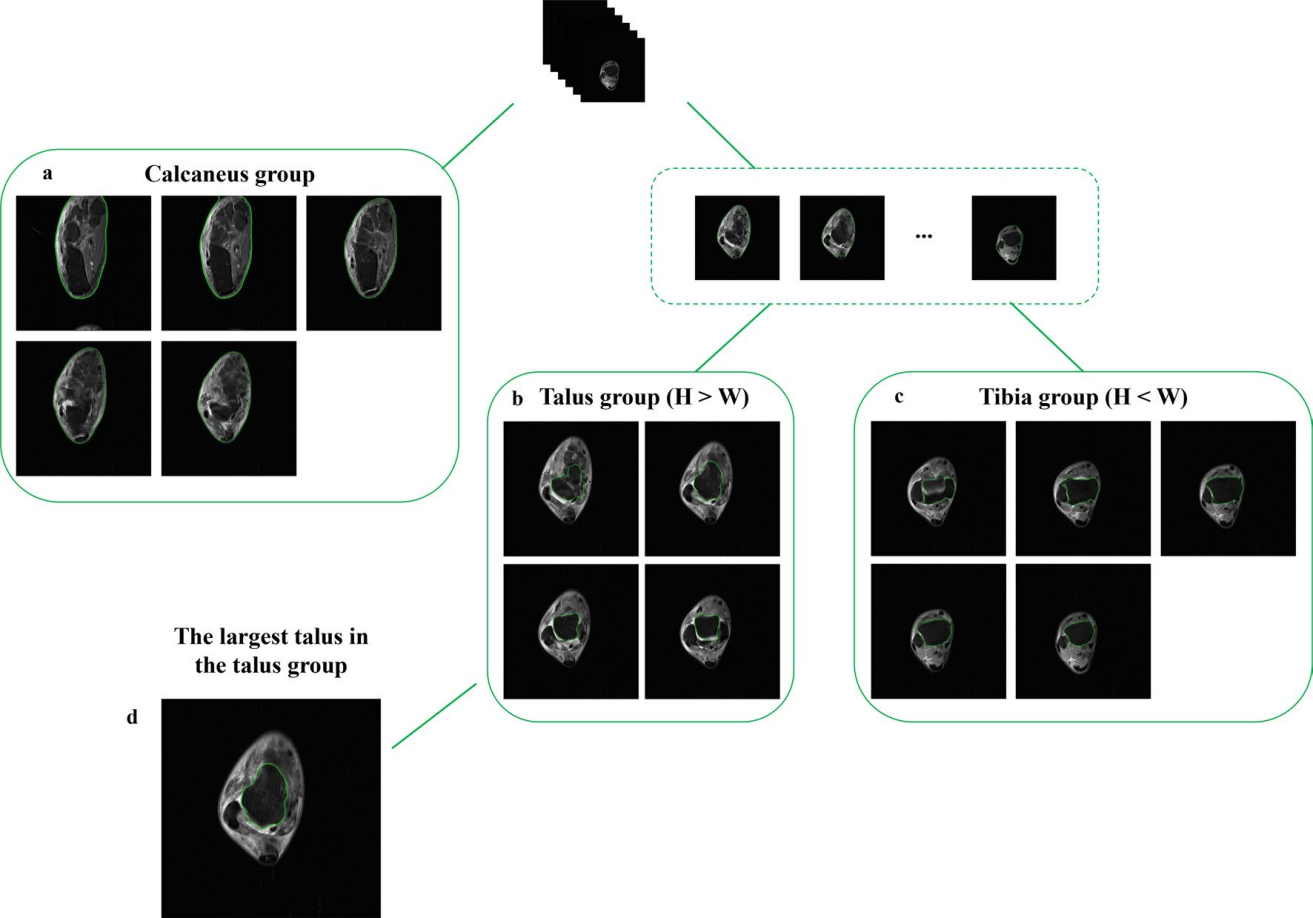
The key slice extraction from cross-sectional T2 weighted images with fat suppression

The scanning range of MRI cross-sectional T2 weighted images with fat suppression for ankle is from the distal tibia to upper calcaneus, which includes the talus, fibula, tibia, calcaneus, and their surrounding soft tissues. The ATFL is connective tissue that connects the talus to the fibula. The bones in the slice where the ATFL presents are mainly talus and fibula. Therefore, based on the tree idea in Fig. 2, the key slice is extracted according to the specific characteristics of bones contained in different slices. Firstly, the outer contours of the foreground in all images are extracted by distance regularized level set evolution (DRLSE) [21]. Adaptive threshold segmentation is used to determine that the outer rectangle with the maximum contour is the initial zero level set. The foreground contour can be obtained by DRLSE. If the number of slices in the whole sequence is represented by n, the first n/3 of the slices with the largest contour perimeter are divided into a batch called calcaneus group, according to the doctor’s instruction. Take the MRI of a patient as an example, Fig. 2a shows the slices of calcaneus group, which mainly includes the calcaneus and its surrounding tissues, but does not include the ATFL. Then, the contour of the bone with the largest area is extracted by DRLSE from the remaining slices, where the pixel area of 20 × 20 in the center of the image is taken as the initial zero level set. The aspect ratio of the minimum bounding rectangle of the contour is calculated, which expressed by $r$. If $r$ is less than 1, the slice will be classified into tibia group as shown in Fig. 2c, in which tibia and its surrounding tissues are present. On the contrary, the slices with $r$ greater than 1 are mainly composed of talus and its surrounding tissues as shown in Fig. 2b, and defined as talus group, from which the key slice containing the ATFL will be found. Since the ATFL exists in the slice with the largest talus area, the slice with the largest contour area in the talus group is selected as the key frame, as shown in Fig. 2d.

#### ATFL localization based on feature points extraction

**Fig. 3:**
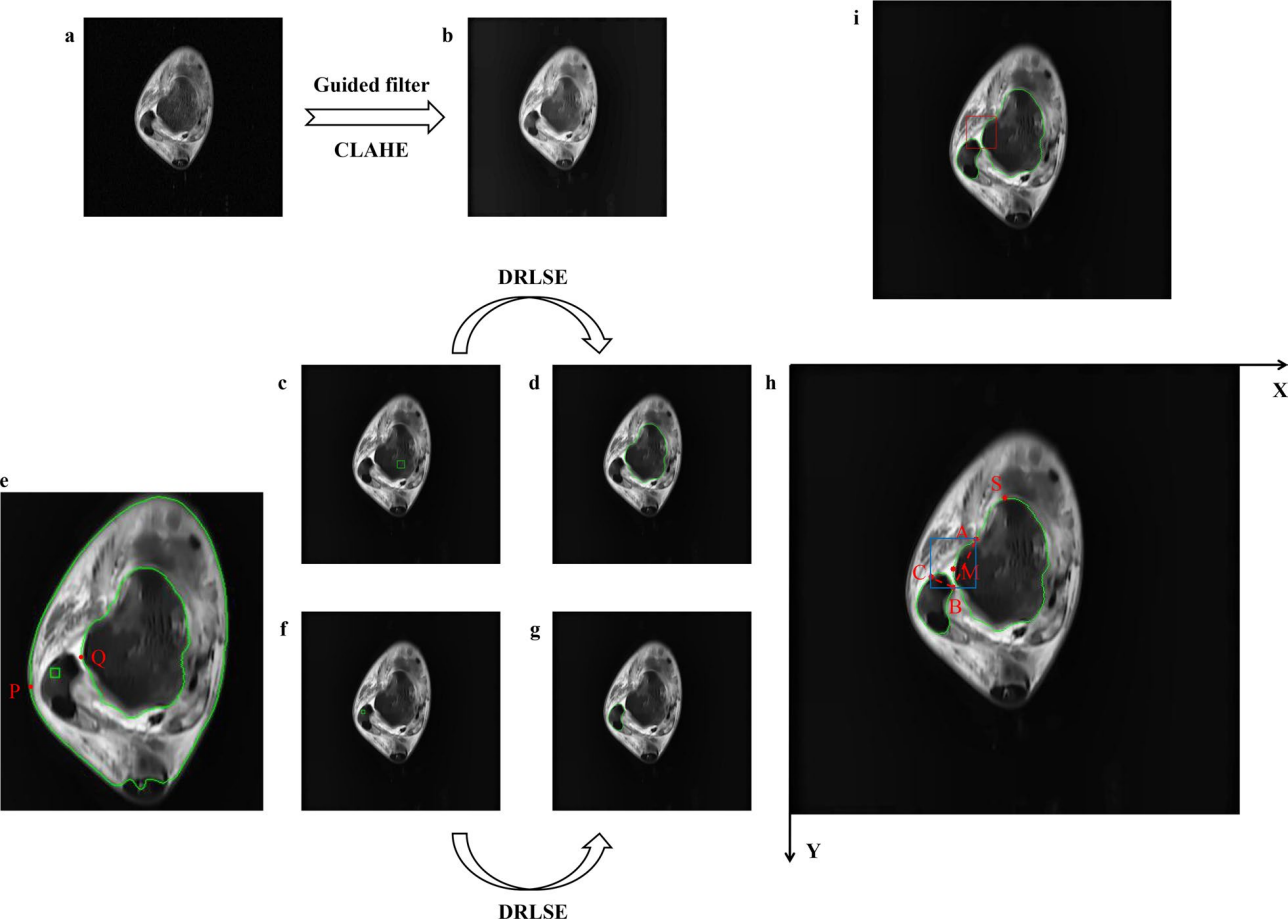
Schematic diagram of ATFL localization process. (**a**. the original image, **b**. the prepocessed image after guided filter and CLAHE, **c**. the initial zero level set of the talus, **d**. the talus contour is obtained by DRLSE, **e**. the initial zero level set of the fibula is determined by the contours of foreground and talus, **f**. the initial zero level set of the fibula, **g**. the fibula contour is obtained by DRLSE, **h**. feature points extraction of ATFL, and **i**. the final result of ATFL localization.)

Due to the characteristics of uneven signal, interrupted continuity, blurred edge and irregular contour in MR image of ligament, it is difficult to locate directly and accurately. Thus, an intelligent localization method based on feature points detection of talus and fibula was proposed in this study. Figure 3 shows the schematic diagram of ATFL localization process, including image preprocessing (Fig. 3a, b), contours extraction of the talus and fibula (Fig. 3c–g), and feature points detection and ATFL localization (Fig. 3h, i).

As shown in Fig. 3a, the original image can easily introduce some noise due to the effect of magnetic field intensity, human thermal noise and other factors in MRI acquisition process, which probably results in the degradation of image quality, or affects the segmentation accuracy [22]. Guided filter [23] and contrast limited adaptive histogram equalization (CLAHE) [24] were used to preprocess the image in this study. The guided filter can be used as an edge-preserving smoothing operator like the popular bilateral filter [25], but it has better behaviors near edges. The preprocessed image is shown in Fig. 3b.

The positioning of ATFL depends on the accurate segmentation of the talus and fibula, as it is attached to the talus on one side and to the fibula on the other. The ATFL arises from the anterior margin of the lateral malleolus, runs below anteromedial, terminates at the neck of the talus and is attached to the lateral margin of the talus aptly in front of the articular surface of the fibula. In view of such anatomical structure characteristics, level set evolution is used to segment the talus and fibula, and the structural feature points of ATFL are determined by calculating the curvature of the contours, whereupon, ATFL can be located and marked. As shown in Fig. 3c, this work used a 20 × 20 pixel area in the center of the image as the initial zero level set. The contour of the talus in Fig. 3d was obtained by DRLSE. However, the initial zero level set of the fibula is related to the contours of foreground and talus. The leftmost boundary point of the foreground contour is marked as P, and the leftmost boundary point of the talus contour is marked as Q, as shown in Fig. 3e. The central 10 × 10 pixel region between P and Q is taken as the initial zero level set, and the fibula contour shown in Fig. 3g is obtained by DRLSE.

Subsequently, feature points are extracted from the contours of the talus and fibula to locate the focus, including the neck of talus, the inner edge of fibula, and the outer edge of fibula. As shown in Fig. 3h, the point in the upper left corner of the image is taken as the origin of coordinates to establish a rectangular coordinate system, where the right direction is taken as the positive direction of X axis, and the downward direction is the positive direction of Y axis. The steps of feature point extraction are as follows.

*Step 1*: Extraction of feature point A.

**Fig. 4:**
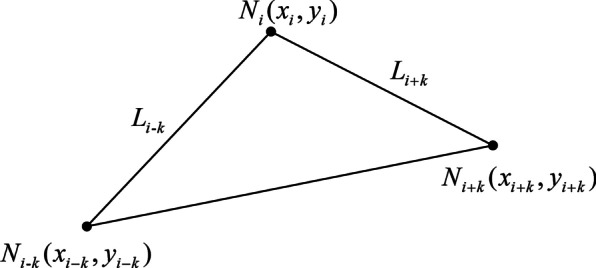
Curvature calculation method of discrete points based on chord length

Among the contour points of the talus, the lateral contour point with the widest width along the X-axis direction is the lateral talus fornix, which is extracted as an auxiliary feature point and marked as M $(x_{M} ,y_{M} )$. The neck of talus (A) is the inflection point between the initial point (S) and the lateral talus fornix (M) in the talus contour, which can be determined by calculating the curvature. The contour extracted by the level set in this work is a discrete digital curve that is composed of a series of discrete points, thus there is no analytical expression. Considering that the curvature of a discrete point is closely related to the relative position of its adjacent points, the method based on chord length is used to calculate the curvature of discrete points. The talus contour points are represented by a set of ordered points that do not coincide, denoted by $N_{i} (x_{i} ,y_{i} )$, where $i = 1,{ 2,} \ldots {, }n$. As shown in Fig. 4, the curvature at the discrete point $N_{i} (x_{i} ,y_{i} )$ is approximately calculated by using point $N_{i - k} (x_{i - k} ,y_{i - k} )$ that is k intervals away from $N_{i}$ forward, and $N_{i + k} (x_{i + k} ,y_{i + k} )$ that is k intervals away from $N_{i}$ backward. Connect points $N_{i}$ and $N_{i - k}$, $N_{i}$ and $N_{i + k}$ to make the contour be a broken line segment. The length of the line segment from point $N_{i - k}$ to $N_{i}$ is denoted by $L_{i - k}$, and the length of the line segment from point $N_{i}$ to $N_{i + k}$ is denoted by $L_{i + k}$. Refer to the curvature calculation method for continuous curves based on arc length, this work used chord length to replace arc length approximatively [26]. The first derivative at point $N_{i}$ can be expressed as

1 $$x_{i}^{^{\prime}} = \frac{{x_{i + k} - x_{i - k} }}{{L_{i - k} + L_{i + k} }}$$

and

2 $$y_{i}^{^{\prime}} = \frac{{y_{i + k} - y_{i - k} }}{{L_{i - k} + L_{i + k} }}$$

The second derivative at point $N_{i}$ can be expressed as

3 $$x_{i}^{^{\prime\prime}} = \frac{{x_{if}^{^{\prime}} - x_{ib}^{^{\prime}} }}{{L_{i - k} + L_{i + k} }}$$

and

4 $$y_{i}^{^{\prime\prime}} = \frac{{y_{if}^{^{\prime}} - y_{ib}^{^{\prime}} }}{{L_{i - k} + L_{i + k} }}$$

where

5 $$x_{if}^{^{\prime}} = \frac{{x_{i + k} - x_{i} }}{{L_{i + k} }}; \, y_{if}^{^{\prime}} = \frac{{y_{i + k} - y_{i} }}{{L_{i + k} }}$$

6 $$x_{ib}^{^{\prime}} = \frac{{x_{i} - x_{i - k} }}{{L_{i - k} }}; \, y_{ib}^{^{\prime}} = \frac{{y_{i} - y_{i - k} }}{{L_{i - k} }}$$

Then, the curvature of point $N_{i}$ is calculated by Eq. (7).

7 $$C_{i} = \frac{{x_{i}^{^{\prime}} y_{i}^{^{\prime\prime}} - x_{i}^{^{\prime\prime}} y_{i}^{^{\prime}} }}{{(x_{i}^{^{\prime}2} + y_{i}^{^{\prime}2} )^{3/2} }}$$

The curvature of each pixel in the talus contour within the range of initial point S and auxiliary point M is calculated according to the above method (k = 10), where the point of maximum curvature is the neck of talus denoted by $A(x_{A} ,y_{A} )$, as shown in Fig. 3h.

*Step 2*: Extraction of feature point B.

The inner edge point of fibula is a inflection point in the fibula contour near the talus side. Therefore, the curvature of every point within this range is calculated, and the coordinate of the minimum curvature point is taken as the inner edge point of fibula denoted by $B(x_{B} ,y_{B} )$.

*Step 3*: Extraction of feature point C.

Connect point A and B, and the vertical segment of line AB through point B intersects the fibula contour on the other side at point C, which is taken as the outer edge point of fibula denoted by $C(x_{C} ,y_{C} )$.

*Step 4*: Localization of the focal area of ATFL.

The positioning results of feature points A, B and C are shown in Fig. 3h. The minimum horizontal and vertical coordinates of feature points A, B and C are taken as the upper-left coordinate and the maximum as the lower-right coordinate, to make a rectangle to locate the focal area of ATFL. Figure 3i shows the final positioning of ATFL.

### Quantitative analysis of MRI characteristics of ATLF

After acquiring the positioning of nidus, the MRI characteristics of ATFL can be quantified. In this study, the first-order gray features and the second-order texture features were extracted to analyse the quantitative features of ATFL.

The normal ATFL shows uniform low signal on MRI, while the injured ATFL shows enhanced high signal due to ligament swelling. Therefore, compared with the normal ATFL, the gray value of injured area is higher in the torn group. The first-order grayscale features can be obtained by counting the frequency distributions of different gray levels in ATFL area. Suppose the total grayscale in the ATFL region is $L$, the gray histogram is defined by

8 $$H(i) = \frac{{n_{i} }}{N},\quad i = 0,1, \ldots ,L - 1$$

where $n_{i}$ is the total number of pixels with gray level $i$, and $N$ is the total number of pixels in ATFL region.

**Fig. 5:**
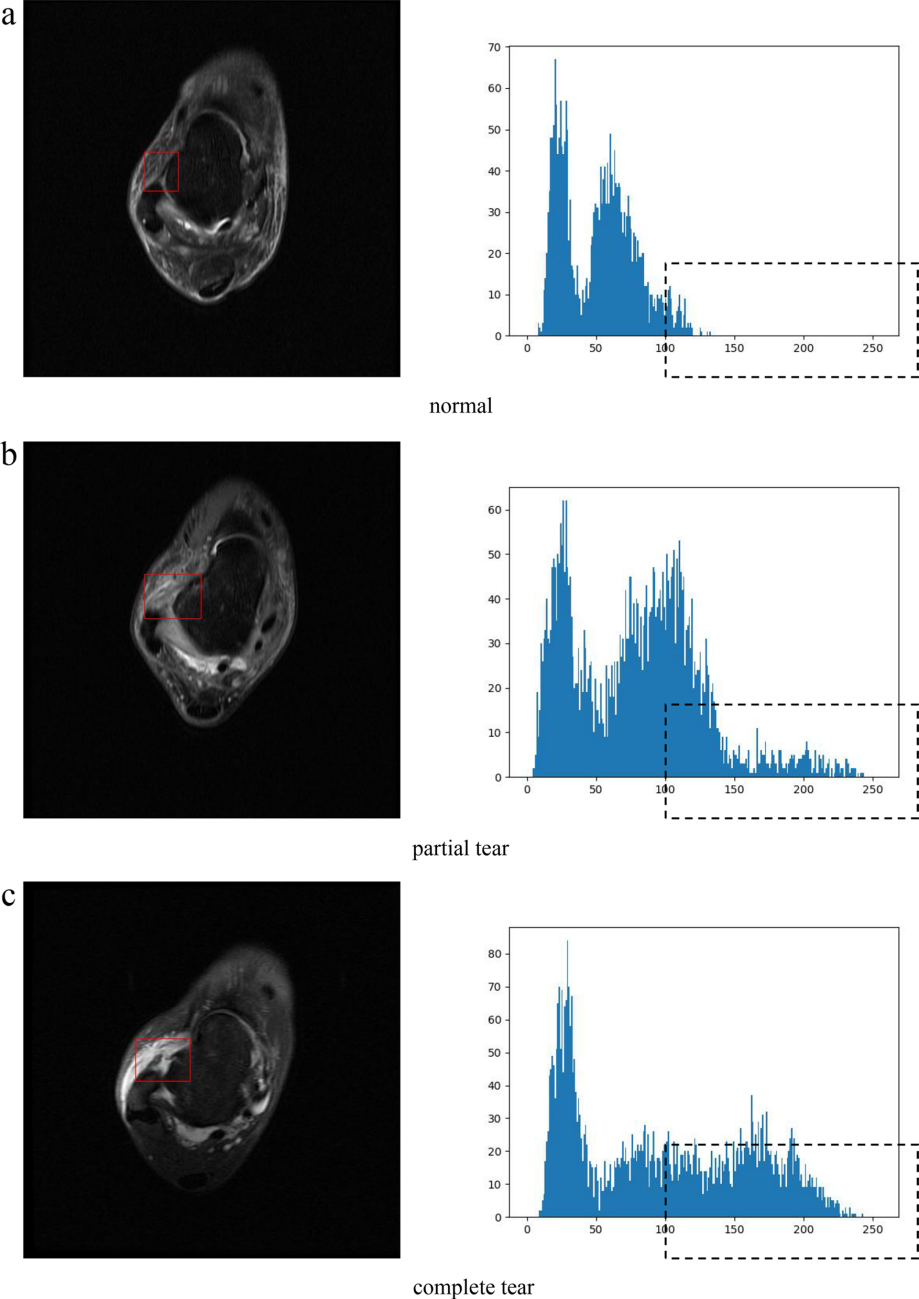
The gray histograms of the ATFL area for normal, partial tear and complete tear

The gray histograms of ATFL area for normal, partial tear and complete tear are shown in Fig. 5, in which the histograms are bimodal, and the gray level corresponding to the right peak of the torn sample is higher than that of the normal sample. According to the part marked by the rectangles in Fig. 5, the torn sample has a larger proportion of high signal pixels than the normal sample. Therefore, the above mentioned proportion of high signal pixels and grayscale corresponding to the right peak are used as the first-order gray features of the ATFL region. Moreover, the conventional histogram statistical features, such as mean value, variance, skewness, kurtosis, energy and entropy, are added into the feature set to form an eight-dimensional first-order gray feature vector. Table 2 shows the calculation formulas of these characteristic parameters.

**Table 2:** The calculation formulas of first-order gray features

Characteristic parameters	Calculation formulas
Mean value	$\mu = \sum\limits_{i = 0}^{L - 1} {iH(i)}$
Variance	$\sigma^{2} = \sum\limits_{i = 0}^{L - 1} {(\mu - i)^{2} H(i)}$
Skewness	$H_{s} = \frac{1}{{\sigma^{3} }}\sum\limits_{i = 0}^{L - 1} {(\mu - i)^{3} H(i)}$
Kurtosis	$H_{k} = \frac{1}{{\sigma^{4} }}\sum\limits_{i = 0}^{L - 1} {(\mu - i)^{4} H(i) - 3}$
Energy	$H_{e} = \sum\limits_{i = 0}^{L - 1} {H(i)^{2} }$
Entropy	$H_{t} = - \sum\limits_{i = 0}^{L - 1} {H(i)\log_{2} (H(i))}$
Proportion of high signal pixels	$P = \frac{1}{N}\sum\limits_{i = L/2}^{L - 1} {n_{i} }$
Grayscale corresponding to the right peak	$Gs = g(\max \{ n_{r} \} , \, r = 50, \ldots ,L - 1)$

This work used texture features based on gray level co-occurrence matrix (GLCM) to quantitatively describe the second-order characteristics of ATFL area, which mainly reflected the variation of spatial gray distribution in ATFL area. Assume that the total number of gray levels of the ATFL image is $L$, then the size of GLCM is $L \times L$, and the formula can be expressed as

9 $$GLCM_{d}^{\theta } (i,j) = |\{ ((r,g),(t,v)):l(r,g) = i,l(t,v) = j\} |$$

where $\forall i,j \in \{ 1,2,...L\}$, $(r,g), \, (t,v) \in L \times L$, and the formula of $(t,v)$ in different angles satisfies

10 $$(t,v) = \left\{ {\begin{array}{*{20}l} {r + d,g} \hfill & {\theta = 0^{ \circ } } \hfill \\ {r + d,g + d} \hfill & {\theta = 45^{ \circ } } \hfill \\ {r,g + d} \hfill & {\theta = 90^{ \circ } } \hfill \\ {r - d,g + d} \hfill & {\theta = 135^{ \circ } } \hfill \\ \end{array} } \right.$$

Let $p(i,j)$ represent the value at $(i,j)$ in GLCM. Then, five texture features including angular second moment, contrast, entropy, inverse differential moment and correlation can be extracted using the GLAM method. Table 3 shows the calculation formulas of these texture parameters.

**Table 3:** The calculation formulas of second-order texture features

Texture parameters	Calculation formulas
Angular second moment	$Asm = \sum\limits_{i = 0}^{L - 1} {\sum\limits_{j = 0}^{L - 1} {[p(i,j)]^{2} } }$
Contrast	$Con = \sum\limits_{i = 0}^{L - 1} {\sum\limits_{j = 0}^{L - 1} {p(i,j)(i - j)^{2} } }$
Entropy	$Ent = - \sum\limits_{i = 0}^{L - 1} {\sum\limits_{j = 0}^{L - 1} {p(i,j)\log p(i,j)} }$
Inverse differential moment	$Idm = \sum\limits_{i = 0}^{L - 1} {\sum\limits_{j = 0}^{L - 1} {\frac{p(i,j)}{{1 + (i - j)^{2} }}} }$
Correlation	$Cor = \frac{1}{{\sigma_{x} \sigma_{y} }}\sum\limits_{i = 0}^{L - 1} {\sum\limits_{j = 0}^{L - 1} {[p(i,j)ij]} - \mu_{x} \mu_{y} }$ where $\mu_{x} = \sum\limits_{i = 0}^{L - 1} {\sum\limits_{j = 0}^{L - 1} {ip(i,j)} }$, $\mu_{y} = \sum\limits_{i = 0}^{L - 1} {\sum\limits_{j = 0}^{L - 1} {jp(i,j)} }$ $\sigma_{x} = \sum\limits_{i = 0}^{L - 1} {\sum\limits_{j = 0}^{L - 1} {(i - \mu_{x} )^{2} p(i,j)} }$,$\sigma_{y} = \sum\limits_{i = 0}^{L - 1} {\sum\limits_{j = 0}^{L - 1} {(j - \mu_{y} )^{2} p(i,j)} }$

The texture parameters of the four directions ($\theta = [0^{ \circ } ,45^{ \circ } ,90^{ \circ } ,135^{ \circ } ]$) are extracted, respectively, so the second-order texture feature vector based on GLCM is 20 dimensions.

### Evaluation of ATFL injury

**Fig. 6:**
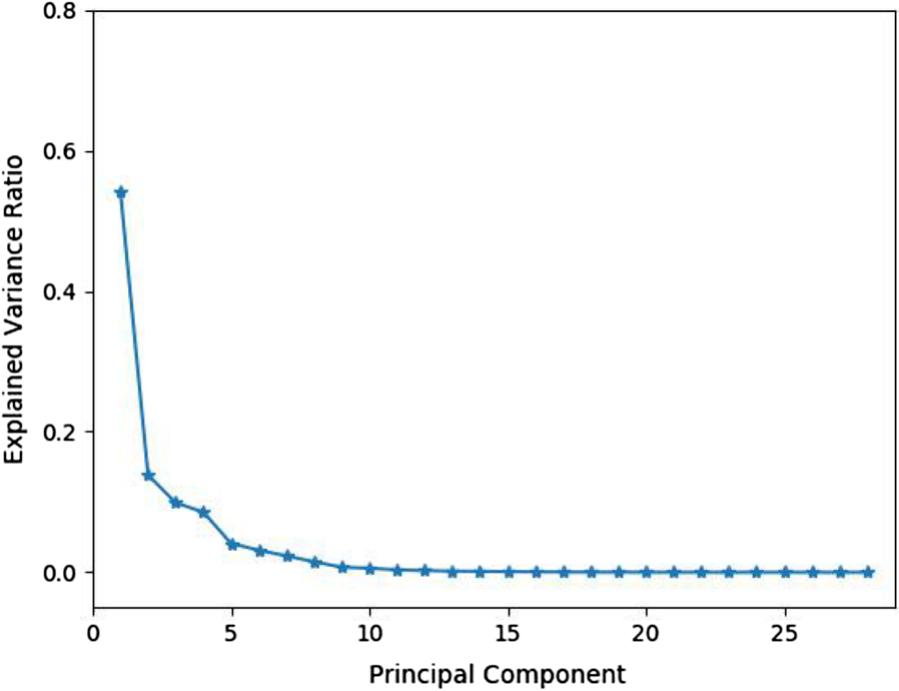
The explained variance ratio of different principal components

The first-order gray features and second-order texture features are merged as a 28-dimensional feature vector. Due to the correlation between different features, it is easy to cause information redundancy. Therefore, this study used principal component analysis (PCA) [27] for feature dimension reduction. Figure 6 shows the explained variance ratio of different principal components, in which the first five principal components contain more than 90% of the original feature information. Thus, these five principal components are selected to replace the original 28-dimensional features, which not only reduces the computational complexity, but also retains most of the effective information in the original features.

Support vector machine (SVM) [28] is used to classify the experimental samples, including 68 normal samples, 64 partial tear samples and 26 complete tear samples. SVM is one of the most successful machine learning methods, which aims to seek the optimal hyperplane with the maximum margin principle. Considering the nonlinearity of experimental data, kernel function is introduced to solve the classification problem. The input of SVM is the principal component features after PCA. Since the dimension of features is small and the number of samples is medium, RBF kernel function is selected to map the features to the high-dimensional space for classification, defined as:

11 $$\kappa (x_{i} ,x_{j} ) = e^{{ - \gamma ||x_{i} - x_{j} ||^{2} }}$$

where $\gamma$ is the only hyper-parameter, $x_{i}$ and $x_{j}$ are sample characteristics.

70% of the sample data are used as training set and 30% as test set. tenfold cross validation [29] is used to divide the training samples into ten parts, in which nine parts are taken as training set, and the corresponding one part is taken as test set in turn. The latest model is selected as the final model to test the samples of the test set. Accuracy, sensitivity, specificity, precision and F1 score are used to evaluate the classification effect of the model. The calculation formulas of these indices are shown in Table 4, where *TP* is true positives, *FP* is false positives, *TN* is true negatives, and *FN* is false negatives.

**Table 4:** The calculation formulas of evaluation indices

Evaluation indices	Calculation formulas
Accuracy	$Acc = \frac{TP + TN}{{TP + FN + FP + TN}}$
Sensitivity	$Sen = \frac{TP}{{TP + FN}}$
Specificity	$Spe = \frac{TN}{{FP + TN}}$
Precision	$\Pr e = \frac{TP}{{FP + TP}}$
F1 score	$F1 = \frac{2*\Pr e*Sen}{{\Pr e + Sen}}$

## Results

### The results of ATFL localization

This experiment used Intel Core i7-7700 K CPU @ 3.80 GHz processor, NVIDIA GeForce RTX 2080TI and operation system based on windows 10 to implement ATFL localization algorithm. PyCharm was used as Integrated Development Environment (IDE), and Python was used as programming language. In order to objectively evaluate the performance of ATFL localization, the positioning accuracy (PA) and Dice Similarity Coefficient (DSC) are used as evaluation index [30]. The closer the value is to 1, the better the positioning effect is. The two metrics are defined as follows:

12 $$PA = \frac{{\left| {\Omega_{1} \cap \Omega_{2} } \right|}}{{\left| {\Omega_{2} } \right|}} \times 100\%$$

13 $$DSC = \frac{{2\left| {\Omega_{1} \cap \Omega_{2} } \right|}}{{\left| {\Omega_{1} } \right| + \left| {\Omega_{2} } \right|}} \times 100\%$$

where $\Omega_{1}$ and $\Omega_{2}$ represent the localization results of proposed method and ground truth, respectively. Table 5 shows the mean values of PA and DSC for normal group, partial tear group and complete tear group. The mean values on PA and DSC of all samples are 87.7% and 77.1%, respectively. Compared with organ segmentation, the accuracy of ATFL positioning in this work is not high enough, due to the larger positioning area, but it does not affect feature extraction and quantitative evaluation in the following. Therefore, the positioning method combined with prior knowledge is reasonable and feasible, which can be a breakthrough in the localization of ligaments.

**Table 5:** The results of ATFL localization

	Normal	Partial tear	Complete tear	Mean
PA (%)	90.7	86.6	85.9	87.7
DSC (%)	77.9	76.7	76.5	77.1

### The results of ATFL injury classification

The computing environments for ATFL injury classification are the same as the localization algorithm. Scikit-learn based on Python was used to train the SVM, where the version of scikit-learn is 0.19.2. By training the SVM model and adjusting the hyper-parameter, the value of $\gamma$ is determined to be 0.5, and the prediction results of 48 samples in the test set are shown in Table 6. Confusion matrix [31] can improve the visualization of classification results, of which the columns represent the predicted results, the rows represent the actual labels, and the diagonal represents the number of accurate classifications per category. As shown in Table 6, the classification accuracy of the model for normal samples is extremely high, reaching 100%, while the accuracy of partial tear and complete tear samples are 70% and 57.1%, respectively. The average accuracy of the entire test set is 81.3%. Furthermore, the average precision is 82%, and the average F1 score is 80%.

**Table 6:** The classification results of the test set for normal, partial tear and complete tear

The actual labels / The predicted results	Normal	Partial tear	Complete tear	Total	Sen (%)
Normal	21	0	0	21	100
Partial tear	4	14	2	20	70
Complete tear	1	2	4	7	57.1
Total/avg	26	16	6	48	81.3

Due to the poor classification effect of partial tear and complete tear group, we combined them into one class called tear group with the consent of doctors. SVM was used again to classify the normal samples and the injury samples. Similarly, 30% of all samples are taken as the test set including 21 normal samples and 27 tear samples. After several training, the value of hyper-parameter $\gamma$ is 0.7, and Table 7 shows the classification results. The average accuracy is 93.8%. For tear group, it gives a good prediction ability with sensitivity of 88.9%, specificity of 100%, precision of 100%, and F1 score of 94.2%. Compared with Table 6, the classification effect of normal and tear is better than that of normal, partial tear and complete tear, which means the feature discrimination of normal group and tear group is stronger, while the discrimination of partial tear and complete tear is worse. It can be concluded that the first-order gray features and the second-order texture features can well explain the differences of MRI lesion area in patients with ATFL injury compared with normal patients.

**Table 7:** The classification results of the test set for normal and tear

The actual labels / The predicted results	Tear	Normal	Total	Sen (%)
Tear	24	3	27	88.9
Normal	0	21	21	100
Total/avg	24	24	48	93.8

## Discussion

In this work, we have developed a novel method for intelligent localization and quantitative evaluation of ATFL injury. The key slice extraction is a significant topic in clinical diagnosis, which can ease the burden on doctors. According to the specific characteristics of organs or tissues in different slices, the key slice was extracted by image segmentation and characteristic analysis. The idea of extracting target images from a series of images can be applied to the diagnosis of other diseases and lesions. Furthermore, in order to solve the difficult problem of direct localization caused by blurred edges and irregular structures of injured ligaments, this work has proposed an indirect method to locate the ATFL by segmenting the talus and fibula with clear contour and detecting their anatomical feature points. Experimental results indicate that the localization method is feasible.

After acquiring the ATFL area, quantitative analysis of MRI characteristics of lesions can be underwent. The first-order gray features and the second-order texture features were extracted to describe the difference between normal and injured ligaments. The normal ATFL shows uniform low signal on MRI, while the injured ATFL shows enhanced high signal due to ligament swelling [11]. The reason is that inflammation causes tissue fluid to seep into ligament area once ligament fibers are partially or completely torn [32]. The first-order grayscale features can be obtained by counting the frequency distributions of different gray levels in the region of ATFL. The normal ATFL shows continuous fibers without tortuosity and regular texture, while the injured ATFL shows broken fibers, tortuous ligament, irregular texture and fuzzy edge. The second-order texture features based on gray level co-occurrence matrix can be obtained to reflect the variation of spatial gray distribution in ATFL area. Compared with qualitative characteristics, quantitative analysis can reduce subjective judgment and make the results more convincing.

In recent years, there has been a significant increase in the number of studies using deep learning technique for purposes such as disease detection and classification, organ and lesion segmentation in the medical imaging fields [33, 34]. With all these developments, the use of deep learning networks to interpret radiological images in musculoskeletal radiology has also become widespread [35, 36]. In terms of assessing ligament injuries, Chang et al. [37] proposed a customized 3D deep learning architecture based on dynamic patch-based sampling, which demonstrated high performance in detection of complete anterior cruciate ligament tears with over 96% test set accuracy. Liu et al. [38] developed a fully automated diagnosis system by using two deep convolutional neural networks to isolate the anterior cruciate ligament on MR images, followed by a classification convolutional neural network to detect structural abnormalities within the isolated ligament. The experimental results verified the feasibility of deep learning method in detection of anterior cruciate ligament tears. Awan et al. [39] used a customized 14 layers ResNet-14 architecture of convolutional neural network with six different directions by using class balancing and data augmentation. The average accuracy for healthy ligament, partial tear and fully ruptured tear had result of 92%. However, deep learning models require large amounts of data to train, on account of the complex structure of networks. The acquired data in this work are very limited, and data amplification technology may cause data redundancy. So deep learning networks are not suitable for the localization and evaluation of ATFL lesions at present. While the proposed method in this study does not need a large number of samples, which greatly reduces the computational expense and the complexity of the model. Features extraction method based on doctors' prior knowledge and image processing algorithm can satisfy the classification problem of small sample data set. Also, the presented method shows good performances in rapid localization, feature quantification and classification of lesions, which has a certain potential value for clinical auxiliary diagnosis.

A potential limitation of our study was that we used a relatively simple model architecture to train the data, instead of convolutional neural network or deep learning. The reason is that the existing deep learning networks are very huge, and need a large number of samples to support. In addition, the training effect depends greatly on the accuracy of labels. In the field of medical imaging processing, the labeling of the lesion area involves a wealth of medical knowledge and experience, coupled with privacy and ethical issues involved in medical data, it is impossible for medical images to be processed by a large number of outsourced personnel like natural images. Thus, considering the limitation of our prepared data and the difficulty of labeling, the training of thousands of network parameters cannot be completed. But we will continue to accumulate samples and labels, so as to use appropriate networks to improve the accuracy of ATFL localization and classification. Further work will focus on more precise classification in acute ATFL injury with different severity. Deep learning networks will be needed to improve the performance of ATFL positioning and evaluation using larger and more varied sets of samples. More importantly, optimizing and generalizing the model to diagnose other ligamentous injury is necessary.

## Conclusion

This study presented a novel method for intelligent localization and quantitative evaluation of ATFL injury, which is helpful for clinical diagnosis. The key slice with ATFL was firstly obtained by extracting the foreground contour and the central bone contour from MRI cross-sectional images. By segmenting the talus and fibula in the key slice and calculating the curvature of their contour pixels, feature points were found to locate ATFL injury area. We have analyzed the first-order gray features and second-order texture features of ATFL area quantitatively, and then classified the normal samples and tear samples. It is concluded that the proposed method gives a good prediction ability for evaluation of ATFL injury, and shows excellent performances in rapid localization, feature quantification and classification of lesions.

## Data Availability

The datasets used and analysed during the current study are available from the corresponding author on reasonable request.

## Abbreviations

ATFL: Anterior talofibular ligament
DRLSE: Distance regularized level set evolution
SVM: Support vector machine
MRI: Magnetic resonance imaging
CLAHE: Contrast limited adaptive histogram equalization
GLCM: Gray level co-occurrence matrix
PCA: Principal component analysis
IDE: Integrated development environment
PA: Positioning accuracy
DSC: Dice Similarity coefficient
